# Effect of Dipeptidyl Peptidase-4 Inhibitors on Bone Metabolism and the Possible Underlying Mechanisms

**DOI:** 10.3389/fphar.2017.00487

**Published:** 2017-07-25

**Authors:** Yinqiu Yang, Chenhe Zhao, Jing Liang, Mingxiang Yu, Xinhua Qu

**Affiliations:** ^1^Department of Endocrinology, Zhongshan Hospital, Fudan University Shanghai, China; ^2^Department of Orthopedics, Shanghai Key Laboratory of Orthopaedic Implants, Shanghai Ninth People's Hospital, Shanghai Jiaotong University School of Medicine Shanghai, China

**Keywords:** DPP-4 inhibitors, fracture risk, bone metabolism, bone formation, bone resorption

## Abstract

Diabetes mellitus has been demonstrated to be closely associated with osteoporosis. Accordingly, hypoglycemic therapy is considered effective in treating metabolic bone disease. Recently, the effects of dipeptidyl peptidase-4 (DPP-4) inhibitors, a new type of antidiabetic drug, on bone metabolism have been widely studied. This review mainly describes the effects of DPP-4 inhibitors on bone metabolism, including their effects on bone mineral density, bone quality, and fracture risk. In addition, the potential underlying mechanisms are discussed. Based on the current progress in this research field, DPP-4 inhibitors have been proved to reduce fracture risk. In addition, sitagliptin, a strong and highly selective DPP-4 inhibitor, showed its beneficial effects on bone metabolism by improving bone mineral density, bone quality, and bone markers. With regard to the potential underlying mechanisms, DPP-4 inhibitors may promote bone formation and reduce bone resorption through DPP-4 substrates and DPP-4-related energy metabolism. Vitamin D and other related signaling pathways also play a role in affecting bone metabolism. Although these assumptions are controversial, they provide a translational pharmacology approach for the clinical use of DPP-4 inhibitors in the treatment of metabolic diseases. Prior to the use of these drugs in clinic, further studies should be conducted to determine the appropriate type of DPP-4 inhibitor, the people who would benefit the most from this therapy, appropriate dose and duration, and the effects of the treatment.

## Introduction

It is well known that diabetes is associated with an increased incidence of osteoporosis, possibly mediated by the effects of insulin deficiency and hyperglycemia on the bone (Achemlal et al., 2005). Many researchers have shown that antidiabetic drugs may affect bone metabolism (Montagnani and Gonnelli, 2013; Jing and Zheng-ping, 2015). Recently, dipeptidyl peptidase-4 inhibitors (DPP-4Is), a new type of antidiabetic drug widely used in clinical therapy, have received attention for their potential use in the treatment of osteoporosis.

DPP-4, also known as adenosine deaminase complexing protein-2 or cluster of differentiation 26 (CD26), is a serine protease expressed on the surface of most cell types. It selectively cleaves alanine and proline from polypeptide substrates, resulting in the inactivation of these substrates, including glucagon-like peptide 1 (GLP-1) and gastric inhibitory polypeptide (GIP) (Green et al., 2006; Idris and Donnely, 2007).

Thus, the mechanism of DPP-4Is involves prolonging the half-life of these incretins (GLP-1 and GIP) and consequently promoting insulin secretion from β-pancreatic cells to improve glucose tolerance (Jing and Zheng-ping, 2015).

In the last decade, DPP-4Is such as vildagliptin, sitagliptin, linagliptin, and saxagliptin have been developed and approved for the treatment of diabetes. In the following review, we summarize the related study progress to date and introduce the potential mechanisms mediating the effects of DPP-4Is on the bone, with the aim of providing a translational pharmacology approach for the clinical application of DPP-4Is in osteoporosis.

## Effect of DPP-4Is on fracture risk

A meta-analysis of 28 randomized clinical trials concluded that treatment with DPP-4Is reduced the risk of fractures when compared with placebo or other treatments (Mantel-Haenszel-odds ratio [HR] 0.60, 95% confidence interval [CI] 0.37–0.99, *P* = 0.045; Monami et al., 2011). A German retrospective analysis revealed that in patients with type 2 diabetes (T2D), the use of DPP-4Is in combination with metformin significantly decreased the risk of developing bone fractures compared to metformin monotherapy (HR = 0.67, 95% CI 0.54–0.84; Dombrowski et al., 2017). Similar results were reported by another nationwide study in South Korea (Choi et al., 2016), which showed the efficacy of DPP-4Is in reducing bone fracture risks, implying their therapeutic potential in osteoporosis.

However, another meta-analysis revealed that there was no difference in the risk of fracture between DPP-4I users and controls (risk ratio 0.95; 95% CI 0.83–1.10; *P* = 0.50) even in subgroup analyses of different types of DPP-4Is, different controls, and different follow-up durations (Fu et al., 2016). This finding was also proved by another meta-analysis reported by Mamza et al. (2016) and a retrospective population-based cohort study (Driessen et al., 2017).

These differences in results may be due to the following reasons: (1) small sample sizes of randomized clinical trials included in the meta-analyses, which may lead to very low fracture incidences in both experimental and control groups; (2) in most of the studies analyzed, the definition of fracture was not clear and various pathological fractures were included, including high-energy fractures; and (3) patients with type 2 diabetes in clinical trials might have used other drugs simultaneously, which may affect the results of the meta-analysis. These problems should be addressed in future studies to provide a translational pharmacology approach for the clinical application of DPP-4Is in osteoporosis.

## Effect of DPP-4Is on bone mineral density (BMD) and bone quality

A cross-sectional study of 744 postmenopausal women in China revealed that participants in the highest quartile of DPP-4 activity had lower BMD in the lumbar spine and femoral neck (*P* < 0.05; Zheng et al., 2015). Another study conducted in obese women also demonstrated that DPP-4 activity negatively correlated with BMD (*R* = −0.288, *P* = 0.038) in the spine (Kim and Cho, 2016), suggesting that the usage of DPP-4Is might improve BMD. However, this finding is still controversial according to a recent study (Carbone et al., 2017).

In animal studies, sitagliptin treatment was reported to improve the vertebral volumetric BMD (Kyle et al., 2011). This effect was also found to be dose-dependent (Cusick et al., 2013). With regard to bone quality, researches conducted in diabetic rats showed that sitagliptin significantly improved cortical bone volume, trabecular architecture, and bone strength (Kyle et al., 2011; Glorie et al., 2014). The results of these studies suggest the possibility that DPP-4I, especially sitagliptin, may be effective in treating osteoporosis and reducing the risk of fractures by improving BMD and bone quality. However, different results were obtained for other kinds of DPP-4I. MK-0626 was reported to have neutral effects on bone quality (Achemlal et al., 2005), whereas saxagliptin showed negative effects (Sbaraglini et al., 2014). Since adequate data on these drugs are still lacking, further studies in both animals and humans are needed to validate these arguments.

## Effect of DPP-4Is on bone turnover markers

### Biochemical markers of bone turnover

An analysis of 124 healthy postmenopausal women revealed a positive correlation between serum DPP-4 activity and parathyroid hormone level. From the results, we speculate that DPP-4Is can decrease parathyroid hormone level, and thus inhibit the release of calcium from the bone to blood and reduce bone destruction (Kim and Cho, 2016). Another study showed that compared to other antidiabetic drugs, DPP-4Is could significantly increase the serum concentration of 25-hydroxy vitamin D3 (25(OH)-D), which has been reported to regulate the concentration of calcium and phosphate in the bloodstream, thereby promoting the growth and remodeling of the bone. The level of 25(OH)-D was also associated with the duration of DPP-4I treatment and strength of DPP-4 inhibitory activity (Barchetta et al., 2016).

### Bone formation markers

A cross-sectional study showed that serum DPP-4 was positively associated with the bone formation markers, bone-specific alkaline phosphatase (ALP) and osteocalcin (Notsu et al., 2016). Moreover, sitagliptin was found to significantly reduce alkaline phosphatase (ALP) level (Kubota et al., 2012; Hegazy, 2015). A non-significant decline in serum osteocalcin was also reported (Hegazy, 2015). However, the underlying reason was not well-explained in these studies.

### Bone resorption markers

In a study of 76 non-obese postmenopausal women, serum DPP-4 activity was found to be correlated with serum carboxy-terminal telopeptide (CTX) levels (*R* = 0.284, *P* = 0.016; Choi et al., 2016). Similar findings were observed in a study conducted in diabetic animals, wherein sitagliptin-treated group had significantly lower serum CTX-I levels (Cusick et al., 2013). Urinary deoxypyridinoline, another bone resorption marker, was also found to be significantly reduced in postmenopausal diabetic women who received sitagliptin (100 mg/day) for 12 weeks (Hegazy, 2015).

However, clinical trials using vildagliptin showed no changes in both bone formation and resorption markers (Bunck et al., 2012). Saxagliptin was found to increase osteoclastic tartrate-resistant acid phosphatase activity (Sbaraglini et al., 2014).

In summary, among the different types of DPP-4Is, only sitagliptin was proved to affect bone turnover markers. This could be because sitagliptin has high inhibitory activity and selectivity for DPP-4 compared to other DPP-4Is. Therefore, to confirm the therapeutic potential of DPP-4Is in metabolic bone diseases, future studies should focus on sitagliptin and other strong DPP-4Is. In addition, extending the follow-up time might help in studying the ameliorating effects of DPP-4Is. The published human and animal studies on the impact of DPP-4I on bone metabolism are described in Table 1.

**Table 1:** Published human and animal researches on the impact of DPP-4Is on bone metabolism.

**References**	**Subjects**	**Methods**	**Main results**
**HUMAN**			
Monami et al., 2011	A meta-analysis includes 28 RCTs in T2D patients	11,880 DPP-4I users vs. 9,175 comparators	Fracture risk: a reduced fracture risk in DPP-4I users (MH-OR 0.60, 95% CI 0.37–0.99, *P* = 0.045)
Dombrowski et al., 2017	1,262 T2D patients with an initial prescription of metformin	4,160 DPP-4I ever users vs. never users (1:1)	Fracture risk: DPP4I use decreases the risk of developing bone fractures (HR = 0.67, 95% CI 0.54–0.84)
Choi et al., 2016	207,558 Subjects with antidiabetes prescriptions	Metformin + DPP4-I vs. metformin+ sulfonylurea vs. control	Fracture risk: the use of DPP-4Is could be associated with decreased risk of fracture
Fu et al., 2016	A meta-analysis includes 62 RCTs of 62,206 T2D patients	DPP-4I users vs. placebo vs. other drugs	Fracture risk: no different risk of fracture (RR, 0.95; 95% CI, 0.83–1.10; *P* = 0.50), even in subgroups using different type of DPP-4 inhibitor, different type of control, and different length of follow-up
Mamza et al., 2016	A meta-analysis includes 51 eligible RCTs of 36,402 participants	37 RCTs: DPP-4 inhibitor vs. placebo (*n* = 23,974),	Fracture risk: no significant association of fracture events with the use of DPP-4 inhibitor when compared with placebo (OR; 0.82, 95% CI 0.57–1.16, *P* = 0.9) or compared against an active comparator (OR; 1.59, 95% CI 0.91–2.80, *P* = 0.9)
		14 RCTs: DPP-4 inhibitor vs. an active comparator. (*n* = 12,428)	
Driessen et al., 2017	328,254 T2D patients with at least one prescription for a non-insulin antidiabetic drug	DPP-4 inhibitor users vs. other antidiabetic drug users	Fracture risk: the use of DPP-4 inhibitors was not associated with risk of any fracture (adjusted hazard ratio [HR] 0.99 [95% confidence interval {CI} 0.93–1.06])
Zheng et al., 2015	744 Postmenopausal women with normal glucose tolerance	High DPP4 activity vs. low DPP4 activity	BMD: Participants in the highest quartile of DPP4 activity had lower BMD (lumbar spine and femoral neck) compared with participants in the lowest quartile (*P* < 0.05)
Kim and Cho, 2016	124 Obese postmenopausal women	The association between BMD and DPP-4 activity	BMD: Serum DPP-4 activity was negatively correlated (*R* = 0.288, *P* = 0.038)
Carbone et al., 2017	1,536 Male and female participants	The association between BMD and DPP-4 activity	BMD: In multivariable adjusted models, there was no association of plasma DPP-4 activity with BMD overall (*p* 0.55 for all) or in gender stratified analyses (*p* 0.23)
Barchetta et al., 2016	295 Consecutive individuals with type 2 diabetes	DPP4-Is(53%) vs. control	25(OH)D level: DPP4-Is-treated participants had significantly higher serum 25(OH)D levels than those undertaking other antidiabetic therapies (18.4 ± 10.7 vs. 14.9 ± 8.6 ng/ml, *p* = 0.004), correlated with the duration and strength of DPP4 inhibitory
Notsu et al., 2016	204 Japanese men with T2DM	The association between serum DPP4 and bone markers (BMD)	• ALP: positively associated with serum DPP-4 • Osteocalcin: positively associated with serum DPP-4
			• Tartrate-resistant acid phosphatase 5b (TRACP-5b): positively associated with serum DPP-4
			• BMD: not changed
Hegazy, 2015	40 Postmenopausal diabetic women	• Metformin (500 mg, bid) vs. sitagliptin (100 mg/d) • duration: 12 weeks	• ALP: the mean serum total ALP was significantly decreased in sitagliptin-treated group
			• Osteocalcin: serum osteocalcin levels were non-significantly decreased gradually by 10% at 12 weeks in sitagliptin-treated group
			• UDPD: urinary DPD decreased significantly and was then maintained at 28% decrease at 12 weeks in sitagliptin-treated group
Kubota et al., 2012	940 Type 2 diabetes mellitus patients	After sitagliptin administration vs. before	ALP: decreased significantly after sitagliptin administration, from 255.3 ± 93.0 IU/L at baseline to 240.3 ± 86.1 IU/L at 4 weeks (*P* < 0.01) and 231.8 ± 78.2 IU/L at 12 weeks (*P* < 0.01)
Bunck et al., 2012	59 Drug-naïve patients with type 2 diabetes	Vildagliptin 100 mg/d for 1 year (*n* = 29) vs. placebo (*n* = 30)	CTX, not changed; ALP, not changed; calcium, not changed
**ANIMALS**			
Kyle et al., 2011	Male and female C57BL/6 mice HFD-fed	Pioglitazone vs. sitagliptin vs. genetic DPP-4 inactivation	• BMD: Sitagliptin treatment significantly improved vertebral volumetric BMD in female mice (not OVX).
			• Bone quality: Sitagliptin significantly improved trabecular architecture and reduced trabecular separation in female mice (not OVX). OVX *Dpp4*^/^ mice exhibited significantly reduced femoral size and mechanics
Cusick et al., 2013	Non-parous female Sprague-Dawley rats	Sitagliptin (100, 300, or 500 mg/kg/day for 12 weeks) vs. blank control group	BMD: BMD generally did not differ significantly between OVX-sitagliptin-treated animals and OVX-vehicle controls. However, there was significantly less BMD loss in lumbar vertebrae with increasing sitagliptin dose
Glorie et al., 2014	64 Male Wistar rats (2 diabetic and two control groups)	Sitagliptin vs. control	• Bone quality: sitagliptin attenuated trabecular bone loss and prevented cortical bone growth stagnation, resulting in stronger femora
			• CTX: the serum levels of the resorption marker CTX-I were significantly lower in sitagliptin-treated group
Gallagher et al., 2014	Male wild type and diabetic muscle-lysine-arginine mice	MK-0626(4 g/kg) vs. control	Bone quality: MK-0626 has neutral effects on cortical and trabecular bone
Sbaraglini et al., 2014	Three-month-old male Sprague-Dawley rats	Saxagliptin(2 mg/kg/day for 3 weeks) vs. control	• Bone quality: a significant decrease in the femoral osteocytic and osteoblastic density of metaphyseal trabecular bone and a decrease in the average height of the proximal cartilage growth plate
			• TRAP: an increase in osteoclastic tartrate-resistant acid phosphatase (TRAP) activity of the primary spongiosa

## Potential mechanisms of DPP-4Is on bone metabolism

**Figure 1:**
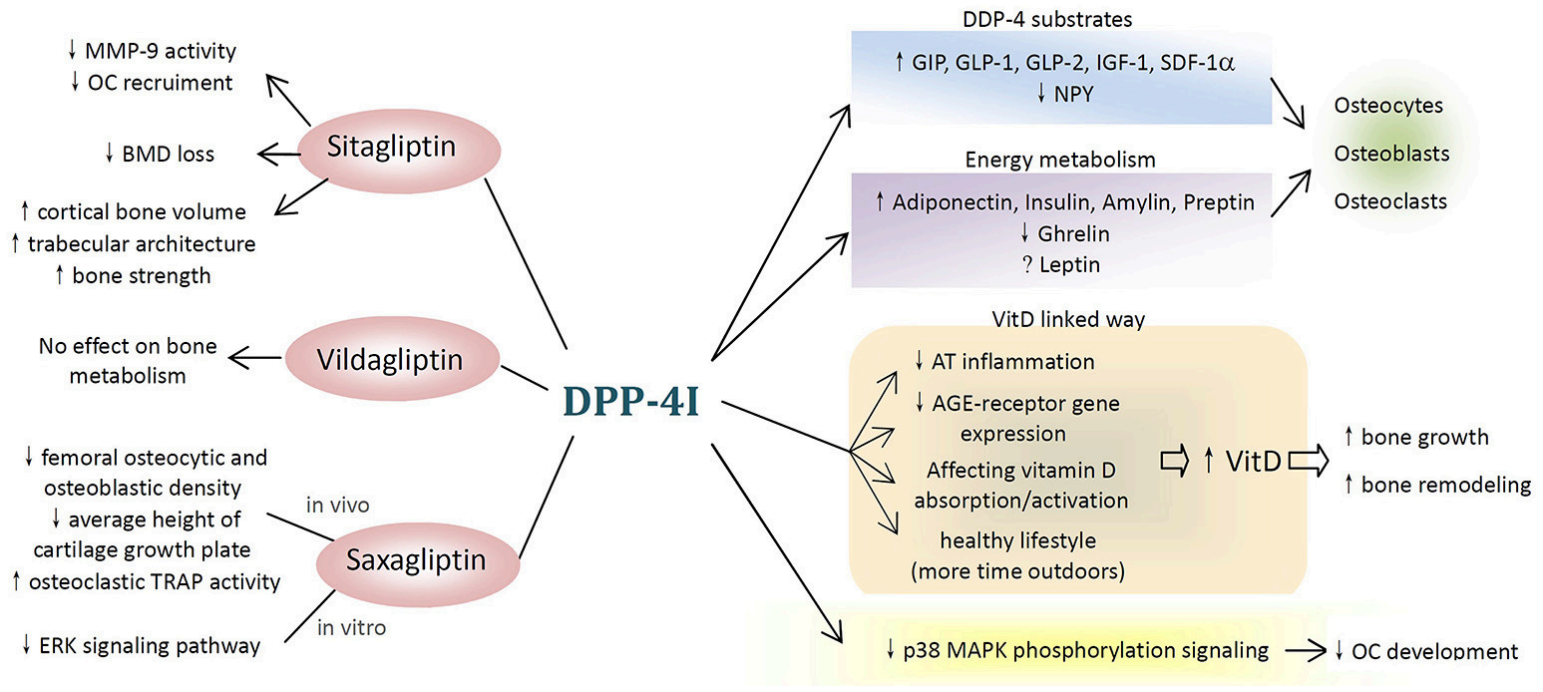
Potential mechanisms of dipeptidyl peptidase-4 inhibitor (DPP-4I) on bone metabolism. (1) Different types of DPP-4Is affect bone metabolism differently. Sitagliptin have beneficial effects on bone resorption, bone mineral density, and bone quality, while vildagliptin shows no effect and saxagliptin negatively affects bone metabolism both *in vivo* and *in vitro*. (2) DPP-4Is attenuate the negative effects of hyperglycemia on the bone. (3) DPP-4Is affect bone cells and bone markers through DPP-4 substrates and DPP-4-related energy metabolism. (4) DPP-4 exerts inhibitory effects on bone metabolism through vitamin D-linked pathway. (5) Other possible pathways (MMP-9, matrix metallopeptidase-9; OC, osteoclast; BMD, bone mineral density; TRAP, tartrate-resistant acid phosphatase; ERK, extracellular signal-regulated kinase; GIP, gastric inhibitory polypeptide; GLP, glucagon-like peptide; SDF-1α, stromal cell-derived factor-1 alpha; NPY, neuropeptide Y; VitD, vitamin D; AT, adipose tissue; AGE, advanced glycation end products; p38 MAPK, p38 mitogen-activated protein kinase).

DPP-4Ts, in particular sitagliptin, showed their therapeutic potential in metabolic bone diseases by reducing fracture risk and improving BMD as well as bone turnover markers. The potential mechanisms underlying these effects are complicated. There are several possible ways through which DPP-4Is may affect bone metabolism, and these are summarized in Figure 1.

### DPP-4Is affect bone metabolism by lowering glucose level

In diabetic patients, insulin deficiency and hyperglycemia seem to play a role in reducing bone formation. Advanced glycation end product (AGE) accumulation or AGE/RAGE (receptors for AGE) axis imbalance directly influences osteoblast activity by decreasing their number and function (Rico et al., 1989; Montagnani and Gonnelli, 2013). Moreover, hyperglycemia negatively affects osteocalcin production and the Wnt signaling pathways through an imbalance in osteoblast/osteoclast activity, leading to bone quality reduction as a global effect (Achemlal et al., 2005; Suzuki et al., 2005).

DPP-4Is can promote insulin secretion from β-pancreatic cells to improve glucose tolerance by prolonging the half-life of GLP-1 and GIP (Jing and Zheng-ping, 2015), and they markedly decrease the negative effects of hyperglycemia on the bone.

### DPP-4Is affect bone metabolism through DPP-4 substrates

#### GIP

DPP-4 can degrade and inactivate polypeptide substrates, such as GIP, by selectively cleaving off alanine and proline (Idris and Donnely, 2007; Jing and Zheng-ping, 2015). Therefore, DPP-4 inhibition can prolong and enhance the effects of GIP on the bone.

In osteoblasts, GIP receptor (GIPR) activation after GIP binding can increase intracellular Ca^2+^ and cyclic adenosine monophosphate (cAMP) concentrations, along with ALP activity and type I collagen mRNA expression (Bollag et al., 2000; Baggio and Drucker, 2007). Moreover, the addition of GIP to cultured osteoblast precursors promotes their differentiation, increases their proliferation, and exhibits an anti-apoptotic activity in multipotent mesenchymal cells in the bone marrow (McIntosh et al., 1996).

GIPR, which is expressed in osteoclasts, is involved in downregulating bone resorption. In mature osteoclasts, GIP inhibits active osteoclast resorptive activity, as assessed by osteoclast pit formation assay, and decreases the expression of osteoclast-differentiation markers, such as the enzymes TRAP and cathepsin K and the M-CSF-receptor (Zhong et al., 2007).

Thus, DPP-4Is may promote bone formation and reduce bone loss by preventing GIP degradation.

#### GLP-1

Similar to GIP, DPP-4Is were reported to inhibit the degradation of GLP-1 by selectively cleaving alanine and proline (Idris and Donnely, 2007; Jing and Zheng-ping, 2015).

In mouse osteoblastic MC3T3-E1 cells, GLP-1 binding to its receptor increased osteocalcin and osteoprotegerin expression, decreased Runt-related transcription factor 2 (Runx2) expression, promoted osteoblast proliferation, and inhibited apoptosis (Nuche-Berenguer et al., 2010). These findings were also proved by an animal study, in which GLP-1 was found to markedly increase osteoblast number, as well as Runx2, ALP, and type I collagen levels in old rats with osteoporosis (Lamari et al., 1996). GLP-1 receptor is also expressed on the surface of osteocytes (Pereira et al., 2015), where it reduced the expression of sclerostin, a protein known to inhibit osteoblastic activity and stimulate catabolic actions on the bone (Kim et al., 2013).

With regard to bone resorption, GLP-1 receptors can be found anchored to the surface of primary osteoclasts. An animal study demonstrated a significant decrease in osteoclast number, CTX-1, and the urinary deoxypyridinoline/creatinine ratio after 16 weeks of GLP-1 use (Henriksen et al., 2003; Glorie et al., 2014). GLP-1 receptors can also be found on the surface of thyroid C cells, which promote the secretion of calcitonin through a cAMP-mediated pathway. Calcitonin inhibits osteoclast activity and prevents the release of calcium from the bone to the blood (Henriksen et al., 2009; Kim and Cho, 2016).

The positive effects of GLP-1 on BMD, bone strength, and bone architecture have been confirmed by animal studies (Achemlal et al., 2005; Mabilleau et al., 2013; Mansur et al., 2015). The various effects of GLP-1 on bone metabolism have been revealed in current studies, pointing out the anabolic effect of DPP-4 inhibition as well as the protective effects against bone loss.

#### Others

Other kinds of DPP-4 substrates also show their individual effects on bone metabolism, which can all be enhanced by DPP-4 inhibition. Stromal cell-derived factor-1 alpha has been found to increase osteoblast proliferation and type-1 collagen expression (Lisignoli et al., 2006). Increased early-stage osteoclast differentiation and reduced osteoclast apoptosis were also observed (Wright et al., 2005). GLP-2 was proved to attenuate bone resorption postprandially in various clinical studies (Haderslev et al., 2002; Henriksen et al., 2003, 2009).

### DPP-4Is affect bone metabolism by affecting energy metabolism

A complex interaction exists between nutrition and bone metabolism, which involves many peptide molecules and adipokines, and this interaction is influenced by DPP-4.

#### Leptin

Leptin is predominantly secreted by adipocytes, and its secretion has been found to be associated with DPP-4 (Lamers et al., 2011). Leptin receptors are expressed in osteoblasts, and binding of leptin to the receptor promotes their proliferation and differentiation (Cornish et al., 2002). It has also been reported to reduce the production of receptor activator of nuclear factor-κ B ligand (RANKL; Cornish et al., 2002) and increase osteoprotegerin expression, thereby reducing osteoclastogenesis (Holloway et al., 2002).

#### Adiponectin

Adiponectin is another peptide specifically secreted by adipocytes. It has been reported to promote the differentiation of osteoblasts as well as stimulate their activity (Wu et al., 2014). In addition, adiponectin suppresses osteoclastic bone resorption (Oshima et al., 2005) and inhibits the differentiation of pre-osteoclasts (Ouchi et al., 2000). DPP-4 inhibition can attenuate the decrease in adiponectin receptor expression in patients with diabetes, thereby promoting bone formation and reducing bone resorption (Sakr, 2013).

#### Ghrelin

DPP-4 inhibition has been found to suppress active ghrelin (McIntosh et al., 1996), an appetite-stimulating hormone produced by the stomach, which stimulates the proliferation and differentiation of osteoblasts (Fukushima et al., 2005) as well as increases bone resorption and bone turnover in a fasted state (Oshima et al., 2005).

### DPP-4Is affect bone metabolism through vitamin D-linked pathway

DPP-4Is significantly increased serum 25(OH)-D concentrations (Barchetta et al., 2016), which in turn led to the growth and remodeling of the bone. The possible mechanisms through which DPP-4Is increase vitamin D level are listed below.

#### DPP-4 modulates adipose tissue (AT) inflammation

AT represents the main site of vitamin D accumulation in humans and is a main target of vitamin D action. DPP-4 is highly expressed in adipocytes and resident macrophages/dendritic cells in inflamed AT (Röhrborn et al., 2015). In diabetic mice, sitagliptin administration has been reported to reduce AT inflammation (Röhrborn et al., 2015). It can be suggested that chronic DPP-4 inhibition modulates AT inflammation and stimulates the activation/mobilization of vitamin D from adipocytes into the bloodstream.

#### DPP-4Is inhibit RAGE expression

The photoconversion of provitamin D into vitamin D might be obstructed by cutaneous AGE accumulation. Notably, direct inhibition of DPP-4 activity by linagliptin was shown to prevent *RAGE* expression in keratinocytes (Schürmann et al., 2012; Krul-Poel et al., 2015; Yamagishi et al., 2015), subsequently improving the local production of vitamin D.

#### DPP-4Is accelerate absorption and activation of vitamin D

Since DPP-4 is also expressed in the intestine, liver, and kidney (Röhrborn et al., 2015), a direct effect of DPP-4Is on vitamin D absorption and activation should be studied. DPP-4I treatment improves glycemic control, which can help lead a healthier lifestyle. Spending more time outdoors can also improve vitamin D levels.

In conclusion, DPP-4Is increase vitamin D level through different mechanisms. It has been well-demonstrated that vitamin D and its metabolites, especially calcitriol, can regulate the concentration of calcium and phosphate in the bloodstream, thereby promoting the growth and remodeling of the bone (Wolf, 2004).

### Other pathways

In addition to the above-mentioned mechanisms, there are other speculations about the mechanism of action of DPP-4Is on bone metabolism that need to be validated. We have listed them below to offer several new possibilities for future studies.

#### Metallopeptidase 9 (MMP-9)-linked pathway

Baertsa et al. reported that sitagliptin could attenuate the increase in MMP-9 activity in patients with diabetes, probably because of its effect on inflammatory response. Therefore, the authors speculated that osteoclast recruitment by MMP-9 might be inhibited by sitagliptin (Baertsa et al., 2015). However, the definite underlying mechanisms remain unclear.

#### P38 mitogen-activated protein kinase (MAPK) phosphorylation pathway

Research suggests that the blockade of CD26 (DPP-4) signaling inhibits the p38 MAPK phosphorylation pathway, which is known to be an important step in early human osteoclast differentiation, and consequently impairs the development of human functional osteoclasts (Nishida et al., 2012, 2014). However, researchers examined the efficacy of thevidagliptin and found no significant inhibitory effect on human osteoclast differentiation or maturation. In addition, vidagliptin did not affect human osteoclast functions (Nishida et al., 2012). These contradictions in results need to be studied further.

#### Extracellular signal-regulated kinase (ERK) signaling pathway

Most studies reported that DPP-4Is positively affect bone metabolism by promoting bone formation and reducing bone loss. However, saxagliptin is so far the only DPP-4I reported to have negative effects on bone metabolism, and its potential underlying mechanisms have been studied.

*In vitro*, saxagliptin was found to inhibit fetal bovine serum-, insulin-, and IGF1-induced ERK phosphorylation and proliferation in both mesenchymal stem cells (MSCs) and MC3T3-E1 pre-osteoblasts in the presence of growth factors. Furthermore, saxagliptin inhibited Runx2 and osteocalcin expression, type-1 collagen production, and mineralization, and increased the expression of peroxisome proliferative activated receptor-gamma in the presence of fetal bovine serum (Sbaraglini et al., 2014).

The negative effects of orally administered saxagliptin on bone metabolism are probably due to the downregulation of the ERK signaling pathway for insulin as well as insulin-like growth factor 1 (IGF-1) in MSCs and the decreased osteogenic potential of these cells (Sbaraglini et al., 2014). However, this study presents certain limitations. The studies were conducted only in healthy rats and *in vitro*, and not in models of insulin resistance or type 2 diabetes. In addition, controlled clinical trials are necessary to verify the effects of saxagliptin on bone adequately.

## Discussion

This review could only partly answer how DPP-4Is affect bone fracture risk, BMD, and bone quality. The potential mechanisms underlying these effects were also partly revealed. Further studies are required before DPP-4Is can be considered for clinical use in metabolic bone disease. We have provided several suggestions about the future research direction as given below:

1. Focus on sitagliptin and other relatively strong DPP-4 inhibitors. Although several kinds of DPP-4Is have been studied, we found that only sitagliptin, a relatively strong DPP-4 inhibitor, showed therapeutic potential in metabolic bone disease. The use of other drugs such as vildagliptin, saxagliptin, and MK-0626 for metabolic bone disease is controversial. Therefore, we suggest that future studies should focus on sitagliptin and other strong DPP-4 inhibitors.
2. More number of clinical trials should be performed in not only diabetic patients, but also other populations. From the study of potential mechanisms, we found that besides lowering glucose level, DPP-4Is might affect bone metabolism through other independent ways. Therefore, we suggest that more number of clinical trials should be performed to prove the effects of DPP-4Is in other populations.
3. The duration and follow-up time should be extended to find chronic changes. DPP-4Is might have chronic effects on bone metabolism, and to prove this, longer duration and follow-up time is required.
4. Dose-related studies to find effective dose with fewer side effects. The effects of DPP-4Is on bone metabolism might be dose dependent (Cusick et al., 2013). However, in most of the current studies, dosage was not regarded as a variable; therefore, we recommend more dose-related studies in the future. Side effects of DPP-4Is should also be taken in consideration.
5. Speculations about the molecular mechanisms of DPP-4 inhibitors in bone physiology should be analyzed further. Although we mentioned many potential mechanisms to explain the relationship between DPP-4Is and bone metabolism in our review, most of them are still speculations lacking adequate evidences.

## Conclusion

Most researchers reported that DPP-4Is have beneficial effects on bone metabolism. Sitagliptin exerted its therapeutic potential in metabolic bone disease by reducing fracture risk and by improving BMD and bone turnover markers. However, studies with vildagliptin and saxagliptin have shown contradictory results.

DPP-4Is can affect bone metabolism through various mechanisms. First, DPP-4 inhibition may promote bone metabolism by lowering glucose level. Second, DPP-4 substrates and DPP-4-related energy metabolism may affect bone metabolism. Third, DPP-4Is might affect bone metabolism through vitamin D-linked and other related signaling pathways. However, the definite underlying mechanisms need to be elucidated further before we can truly provide a translational pharmacology approach for the clinical application of DPP-4Is for osteoporosis.

## Author contributions

All authors listed have made a substantial, direct and intellectual contribution to the work, and approved it for publication.

### Conflict of interest statement

The authors declare that the research was conducted in the absence of any commercial or financial relationships that could be construed as a potential conflict of interest.

## Abbreviations

DPP-4: dipeptidyl peptidase-4
MSCs: mesenchymal stem cells
ERK: extracellular signal-regulated kinase.
